# A network‐based predictive gene expression signature for recurrence risks in stage II colorectal cancer

**DOI:** 10.1002/cam4.2642

**Published:** 2019-11-14

**Authors:** Wen‐Jing Yang, Hai‐Bo Wang, Wen‐Da Wang, Peng‐Yu Bai, Hong‐Xia Lu, Chang‐He Sun, Zi‐Shen Liu, Ding‐Kun Guan, Guo‐Wang Yang, Gan‐Lin Zhang

**Affiliations:** ^1^ Department of Oncology Beijing Hospital of Traditional Chinese Medicine Capital Medical University Beijing China; ^2^ Department of Biochemistry and Molecular Biology Capital Medical University Beijing China; ^3^ Department of Anorectal Surgery Shanxi Cancer Hospital Taiyuan China; ^4^ Department of Gastroenterology Shanxi Cancer Hospital Taiyuan China

**Keywords:** bioinformatics analysis, colorectal cancer, recurrence mechanisms, recurrence risks

## Abstract

The current criteria for defining the recurrence risks of stage II colorectal cancer (CRC) are not robust; therefore, we aimed to explore novel gene signatures to predict recurrence risks and to reveal the underlying mechanisms of stage II CRC. First, the gene expression profiles of 124 patients with stage II CRC from The Cancer Genome Atlas (TCGA) database were obtained to screen differentially expressed genes (DEGs). A total of 202 DEGs, including 128 upregulated and 74 downregulated, were identified in the recurrence group (n = 24) compared to the nonrecurrence group (n = 100). Furthermore, the top 5 DEGs (*ZNF561*, *WFS1*, *SLC2A1*, *MFI2*, and *PTGR1*) were identified by random forest variable hunting, and four (*ZNF561*, *WFS1*, *SLC2A1*, and *PTGR1*) were selected to create a four‐gene recurrent model (GRM), with an area under the curve (AUC) of 0.882 according to the receiver operating characteristic curve, and the robust diagnostic effectiveness of the GRM was further validated with another gene expression profiling dataset (GSE12032), with an AUC of 0.943. The diagnostic effectiveness of the GRM regarding recurrence was associated with poor disease‐free survival in all stages of CRC. In addition, gene ontology functional annotation and Kyoto Encyclopedia of Genes and Genomes pathway enrichment analyses revealed 18 enriched functions and 6 enriched pathways. Four genes, *ABCG2*, *CACNA1F*, *CYP19A1*, and *TF*, were identified as hub genes by the protein‐protein interaction network, which further validated that these genes were correlated with a poor pathologic stage and overall survival in all stages of CRC. In conclusion, the GRM can effectively classify stage II CRC into groups of high and low risks of recurrence, thereby making up for the prognostic value of the traditional clinicopathological risk factors defined by the National Comprehensive Cancer Network guidelines. The hub genes may be useful therapeutic targets for recurrence. Thus, the GRM and hub genes could offer clinical value in directing individualized and precision therapeutic regimens for stage II CRC patients.

## Graphical abstract

This study finds indicators for predicting postoperative recurrence in patients with stage II colorectal cancer (CRC). Moreover, the gene recurrent model and hub genes potentially offer clinical values in directing individualized and precision therapeutic regimen be selection for stage II CRC.

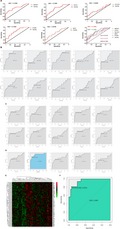

## 1 INTRODUCTION

Colorectal cancer (CRC) is the third most frequent malignant tumor and the fourth leading cause of cancer‐related death worldwide.^1^ Survival and treatment in CRC are primarily dependent on the tumor stage at diagnosis. Radical resection of the tumor lesion is the foundation treatment for stage II CRC. However, postsurgery, 25%‐30% of stage II CRC patients develop recurrence within 5 years,^2^ contributing to mortality. To improve the survival of these patients, the American Society of Clinical Oncology, the National Comprehensive Cancer Network (NCCN) and the European Society for Medical Oncology recommend adjuvant chemotherapy for high‐risk patients with stage II CRC, and the risks are primarily defined by clinicopathological features, such as tumor size, the number of lymph nodes investigated, poorly differentiated histology, tumor perforation (T4), bowel obstruction and perforation, positive resection margins, and lymphatic and venous invasion.^3^

Unfortunately, using these abovementioned criteria, we found that a portion of low‐risk patients also experienced recurrence after the operation.^4^ On the other hand, is there a portion of patients who were overtreated according to the abovementioned criteria? This concern highlights the lack of available biomarkers that can help detect the genuine high‐risk factors of recurrence for stage II CRC, which can improve the treatment accuracy of these patients.

In recent years, rapid technological breakthroughs of genome‐wide sequencing have provided researchers with large expression datasets. With the popularization of big data analysis and the progress of bioinformatics technology, a series of biomarkers was identified for predicting the recurrence, metastasis, chemosensitivity, and prognosis of CRC. Moreover, the complex recurrence and metastatic processes of polygenic network collaboration were also analyzed by bioinformatics tools.^5^, ^6^, ^7^ Practice suggested that the prediction value of a single biomarker was usually unfavorable, and multiple biomarkers jointly confirmed to be efficient and reliable.^8^, ^9^

In this study, we performed bioinformatical analyses based on high‐throughput RNA sequencing of CRC from the The Cancer Genome Atlas (TCGA) (http://cancergenom-e.nih.gov/) to gain a panoramic view of expression patterns between recurrence and nonrecurrence patients with stage II CRC. Furthermore, differentially expressed genes (DEGs) were used to establish a model to predict the recurrence risk (gene recurrent model [GRM]) by random forest sequencing, and excellent diagnostic effectiveness was shown by receiver operating characteristic (ROC) curve analysis. Then, another gene expression profiling dataset was extracted from the GEO (GSE12032) to further validate the robust diagnostic effectiveness of the GRM. In addition, gene ontology (GO), Kyoto Encyclopedia of Genes and Genomes (KEGG), protein‐protein interaction (PPI) network analyses, and hub gene selection were adopted to jointly analyze the underlying mechanism of recurrence. Finally, the results we obtained might be meaningful in guiding clinical practice and understanding the recurrence mechanisms of stage II CRC.

## 2 MATERIALS AND METHODS

### 2.1 Data collection

The RNA‐Seq dataset of CRC, which includes the whole human transcriptome sequencing dataset and corresponding survival profiles, was downloaded from the TCGA database. All the data in the dataset used were pathological stage II (T_3‐4_N_0_M_0_) without postoperative adjuvant therapy and were followed up for at least 2 years. According to recurrence, the samples were divided into a recurrence group and a nonrecurrence group.

### 2.2 Data preprocessing and the identification of DEGs

edgeR is an R package used for the analysis of DEGs^10^ and was used in our study to screen the DEGs between the recurrence and nonrecurrence groups. The DEGs were identified with the following criteria: fold change |(FC)| ≥2 and *P* value <.01.

### 2.3 Random forest sequencing

Random forest is a popular classification and regression method that has proven powerful for various prediction problems in biological studies. The mean decrease in gini (MDG), which is involved in the random forest algorithm, is used to rank the important indexes with DEGs. The MDG provides ways to quantify which index contributes most to classification accuracy. A higher MDG indicates that the degree of impurity arising from the category could be reduced farthest by one variable and thus suggests an important associated index. We divided our data into training (66%, n = 82) and testing (34%, n = 42) datasets by using the randomForest package of R software (http://www.r-project.org).^11^ We used the training dataset to develop the random forest model and then tested the model's performance with the testing dataset. The specific random forest model parameters were as follows: max features: auto, n estimators: 5000, min sample leaf: 1, and number of variables tried at each split: 2.

### 2.4 ROC analysis of the select recurrence‐related genes

Receiver operating characteristic analyses are commonly used to evaluate the performance of disease diagnosis. In our study, the four genes selected from the top 5 genes ranked by the MDG were used as biomarkers for detecting recurrence and for constructing a recurrence risk model. The area under the curve (AUC) was used to demonstrate the accuracy of an individual gene and joint genes for predicting recurrence.

### 2.5 External exploration of the diagnostic effectiveness of the GRM

To further validate the diagnostic effectiveness of the GRM, another gene expression profiling dataset was extracted from the GEO (GSE12032) for analysis. GEO2R, a web tool that was applied to screen the DEGs by comparing two groups of samples in a GEO series, was applied to identify the DEGs between the recurrence and nonrecurrence groups of stage II CRC patients following the criteria fold change |(FC)| ≥1.5 and *P* value <.05. The patients with missing values were excluded. Furthermore, the identified DEGs were ranked by the MDG in the random forest algorithm, and the top 10 genes were selected. In addition, the diagnostic effectiveness of the GRM was validated by ROC analyses, and the AUC was used to demonstrate the accuracy of the GRM for predicting recurrence.

### 2.6 Further exploration of the clinical values of the GRM

As the four genes in the GRM achieved robust predictive values of recurrence in stage II CRC and the risk of recurrence always affected disease‐free survival (DFS), we further validated the four genes by investigating their expression and relevance to DFS in all stages of CRC between the recurrence and nonrecurrence groups. Kaplan‐Meier survival curves were constructed using the expressions of the four genes from the TCGA transcriptional profiles as a threshold and compared by log‐rank analysis. All analyses were performed with GraphPad Prism 5.0 and SPSS version 19.0 (IBM), and *P* < .05 was considered statistically significant.

### 2.7 Functional annotation and pathway enrichment analyses

The Database for Annotation, Visualization, and Integrated Discovery (DAVID) v.6.8 (https://david.ncifcrf.gov)^12^ was used to perform GO^13^ functional annotation and KEGG^14^ pathway enrichment analyses. The human genome was selected as the background list parameter, and a *P* value <.05 was set as statistically significant.

### 2.8 PPI network analysis and hub gene selection

The Search Tool for the Retrieval of Interacting Genes (STRING, http://www.string-db.org/) database was used to construct a PPI network. An interaction score >0.4 was considered statistically significant. Furthermore, the result of the PPI network was imported into the Cytoscape plugin to create network visualizations and subjected to centrality values analysis with CentiScaPe 2.2.

### 2.9 Further exploration of the clinical values of the four hub genes

As the four hub genes that promote recurrence always highly correlated with pathologic stage and affected overall survival (OS), we further validated the four hub genes by investigating their expression in different pathologic stages and relevance to OS in all stages of CRC. Kaplan‐Meier survival curves were constructed using the expressions of the four hub genes from the TCGA transcriptional profiles as a threshold and compared by log‐rank analysis. All analyses were performed with GraphPad Prism 5.0 and SPSS version 19.0, and *P* < .05 was considered statistically significant.

## 3 RESULTS

### 3.1 Identification of the DEGs

**Figure 1:**
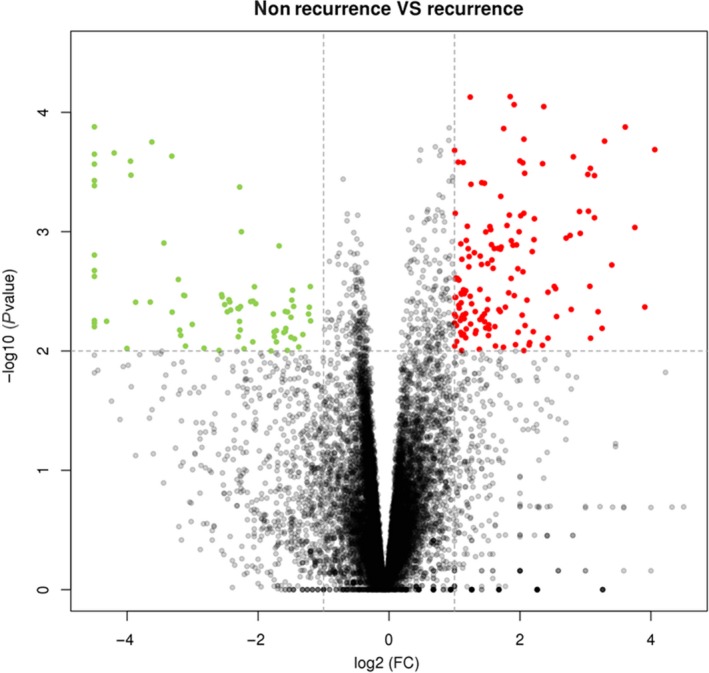
Volcano plot of the DEGs between recurrence and nonrecurrence groups of stage II CRC. The x‐axis indicates the log2 fold change in gene expression, which was defined as the ratio of normalized value of gene expression detected in stage II CRC between recurrence and nonrecurrence groups. The y‐axis indicates the adjusted *P* values plotted in −log10. Red dots highlight genes upregulated in recurrence group (fold change >2, *P* value <.01). Green dots highlight genes downregulated in nonrecurrence group (fold change >2, *P* value <.01). CRC, colorectal cancer; DEGs, differentially expressed genes

Information on the 124 patients who met our research criteria was obtained from the TCGA database. After a 2‐year follow‐up, 24 patients experienced recurrence, and 100 patients did not experience recurrence. Moreover, 202 DEGs, including 128 upregulated and 74 downregulated, were identified in the recurrence group compared to the nonrecurrence group (Figure 1).

### 3.2 Acquisition of the classified DEGs by the random forest classifier

The randomForest package in R, which performs well in predicting whether variables are noise or not and evaluates the importance of the variable, was used. The 202 DEGs were ranked by the MDG with the random forest method, and the top 5 DEGs (*ZNF561*, *WFS1*, *SLC2A1*, *MFI2*, and *PTGR1*) were selected for construction of the ROC curve (Table 1).

**Table 1:** The TCGA top 5 DEGs for ROC construction were ranked by MDG with random forest method

Gene	Description	Mean Decrease Gini (MDG)
*ZNF561*	Zinc finger protein 561	17.608
*WFS1*	Wolframin ER transmembrane glycoprotein	13.193
*SLC2A1*	Solute carrier family 2 member 1	11.726
*MFI2*	Melanotransferrin	9.878
*PTGR1*	Prostaglandin reductase 1	9.510

Abbreviations: DEGs, differentially expressed genes; ROC, receiver operating characteristic; TCGA, The Cancer Genome Atlas.

### 3.3 Construction of a four‐GRM and further validation

**Figure 2:**
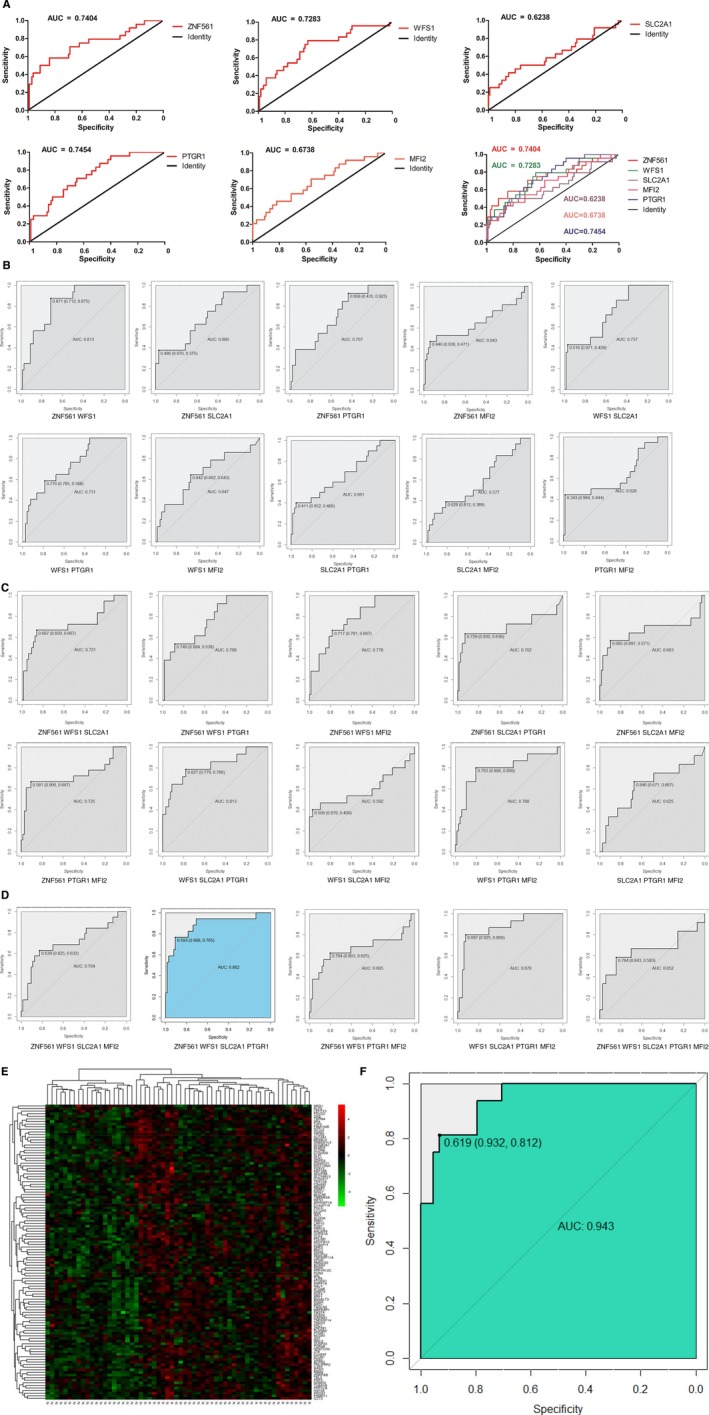
ROC curves of the top 5 DEGs sorted by AUC. Red line represents the sensitive curve, while black represents the identify line. The x‐axis indicates false positive rate, which is presented as “Specificity (1 − Sensitivity)”. The *y*‐axis indicates true positive rate, which is presented as “Sensitivity”. A, The individual diagnostic efficiency of *ZNF561*, *WFS1*, *SLC2A1*, *MFI2*, and *PTGR1*. B, The joint diagnostic efficiency of the combinations of the two DEGs in the top 5. C, The joint diagnostic efficiency of the combinations of the three DEGs in the top 5. D, The joint diagnostic efficiency of the combinations of the four or five DEGs in the top 5. E, Heatmap of the DEGs between recurrence and nonrecurrence groups of stage II CRC. Each column showed patient samples. N represented nonrecurrence. R represented recurrence. A hierarchical clustering analysis was performed, and patient information based on the expression of DEGs was mapped. Red represented upregulated genes. Green represented downregulated genes. F, The diagnostic efficiency of GRM in GEO date. AUC, area under the curve; CRC, colorectal cancer; DEGs, differentially expressed genes; GRM, gene recurrent model; ROC, receiver operating characteristic

The ROC curve defined an optimal threshold to predict the recurrence risk of stage II CRC, and the AUC values of the ROC for *ZNF561*, *WFS1*, *SLC2A1*, *MFI2*, and *PTGR1* were 0.7404, 0.7283, 0.6238, 0.6738, and 0.7454, respectively (Figure 2A). To elevate the prediction efficiency, we explored the combination of two, three, four and five DEGs. The AUC values of the ROC curve with the combination of two DEGs for *ZNF561*+*WFS1*, *ZNF561*+*SLC2A1*, *ZNF561*+*PTGR1*, *ZNF561*+*MFI2*, *WFS1*+*SLC2A1*, *WFS1*+*PTGR1*, *WFS1*+*MFI2*, *SLC2A1*+*PTGR1*, *SLC2A1*+*MFI2*, and *PTGR1*+*MFI2* were 0.813, 0.680, 0.707, 0.643, 0.737, 0.731, 0.647, 0.661, 0.577, and 0.628, respectively (Figure 2B). The AUC values of the ROC curve with the combination of three DEGs for *ZNF561*+*WFS1*+*SLC2A1*, *ZNF561*+*WFS1*+*PTGR1*, *ZNF561*+*WFS1*+*MFI2*, *ZNF561*+*SLC2A1*+*PTGR1*, *ZNF561*+*SLC2A1*+*MFI2*, *ZNF561*+*PTGR1*+*MFI2*, *WFS1*+*SLC2A1*+*PTGR1*, *WFS1*+*SLC2A1*+*MFI2*, *WFS1*+*PTGR1*+*MFI2*, and *SLC2A1*+*PTGR1*+*MFI2* were 0.721, 0.768, 0.778, 0.702, 0.663, 0.725, 0.813, 0.592, 0.788, and 0.625, respectively (Figure 2C). The AUC values of the ROC curve with the combination of four DEGs for *ZNF561*+*WFS1*+*SLC2A1*+*MFI2*, *ZNF561*+*WFS1*+*SLC2A1*+*PTGR1*, *ZNF561*+*WFS1*+*PTGR1*+*MFI2*, and *WFS1*+*SLC2A1*+*PTGR1*+*MFI2* were 0.704, 0.882, 0.665, and 0.870, respectively (Figure 2D). The AUC value of the ROC curve with the combination of five DEGs for *ZNF561*+*WFS1*+*SLC2A1*+*PTGR1*+*MFI2* was 0.652 (Figure 2D). Among them, the four‐gene signature (*ZNF561*, *WFS1*, *SLC2A1*, and *PTGR1*), with an AUC of 0.882, exhibited the best performance for predicting recurrence and showed remarkable sensitivity and specificity when the cutoff value was 0.593 (Figure 2D).

The transcriptome profiling data of 92 patients with stage II CRC in the GSE12032 dataset, which includes 30 patients with recurrence and 62 patients without recurrence, were collected. After excluding the patients with missing values, we obtained 60 patients, including 16 patients with recurrence and 44 patients without recurrence. Furthermore, 113 DEGs (63 upregulated DEGs and 50 downregulated DEGs) were identified in the recurrence group compared to the nonrecurrence group (Figure 2E). In addition, all the genes in the GRM were included in the top 10 DEGs that were ranked by the MDG with the random forest classifier (Table 2). These genes exhibited robust performance for predicting recurrence, with an AUC of 0.943, and showed remarkable sensitivity and specificity when the cutoff value was 0.619 (Figure 2F).

**Table 2:** The top 10 DEGs of GSE12032 were ranked by MDG with random forest method

Gene	Description	Mean Decrease Gini (MDG)
*ABCB5*	Zinc finger protein 561	1.405
*SLC2A1*	Solute carrier family 2 member 1	1.300
*CLDN6*	Claudin 6	0.924
*ZNF561*	Zinc finger protein 561	0.808
*HIST1H4H*	Histone cluster 1 H4 family member h	0.773
*TMEM63A*	Transmembrane protein 63A	0.725
*PTGR1*	Prostaglandin reductase 1	0.615
*BCAR3*	BCAR3 adaptor protein, NSP family member	0.551
*WFS1*	Wolframin ER transmembrane glycoprotein	0.517
*C3orf42*	Long intergenic nonprotein coding RNA 852	0.475

Abbreviation: DEGs, differentially expressed genes.

### 3.4 The clinical values of the four genes in the GRM for all stages of CRC

**Figure 3:**
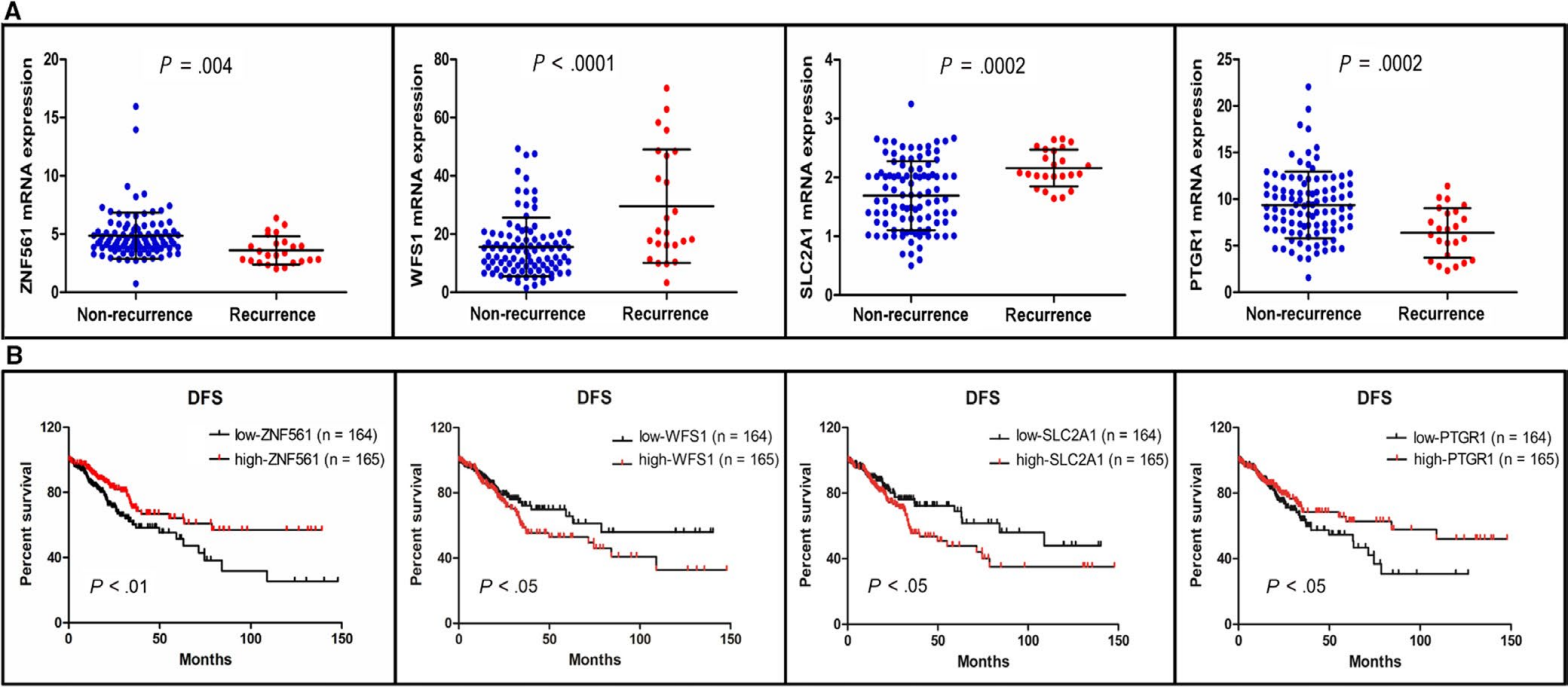
The clinical value of the top 4 genes in all stage CRC. A, Validation of the gene expression levels of *ZNF561*, *WFS1*, *SLC2A1*, and *PTGR1* between recurrence and nonrecurrence patients. B, Disease‐free survival analysis of the top 4 genes. CRC, colorectal cancer

The expression levels of the four genes in the GRM between the recurrence and nonrecurrence groups were significantly different, and the *P* values for *ZNF561*, *WFS1*, *SLC2A1*, and *PTGR1* were .004, <.0001, .0002, and .0002, respectively (Figure 3A). The survival analysis indicated that low *ZNF561*, *PTGR1* expression and high *WFS1*, *SLC2A1* expression were associated with poor DFS, with *P* values of <.01, <.05,<.05, and <.05, respectively (Figure 3B).

### 3.5 Functional annotation and pathway enrichment analyses

**Figure 4:**
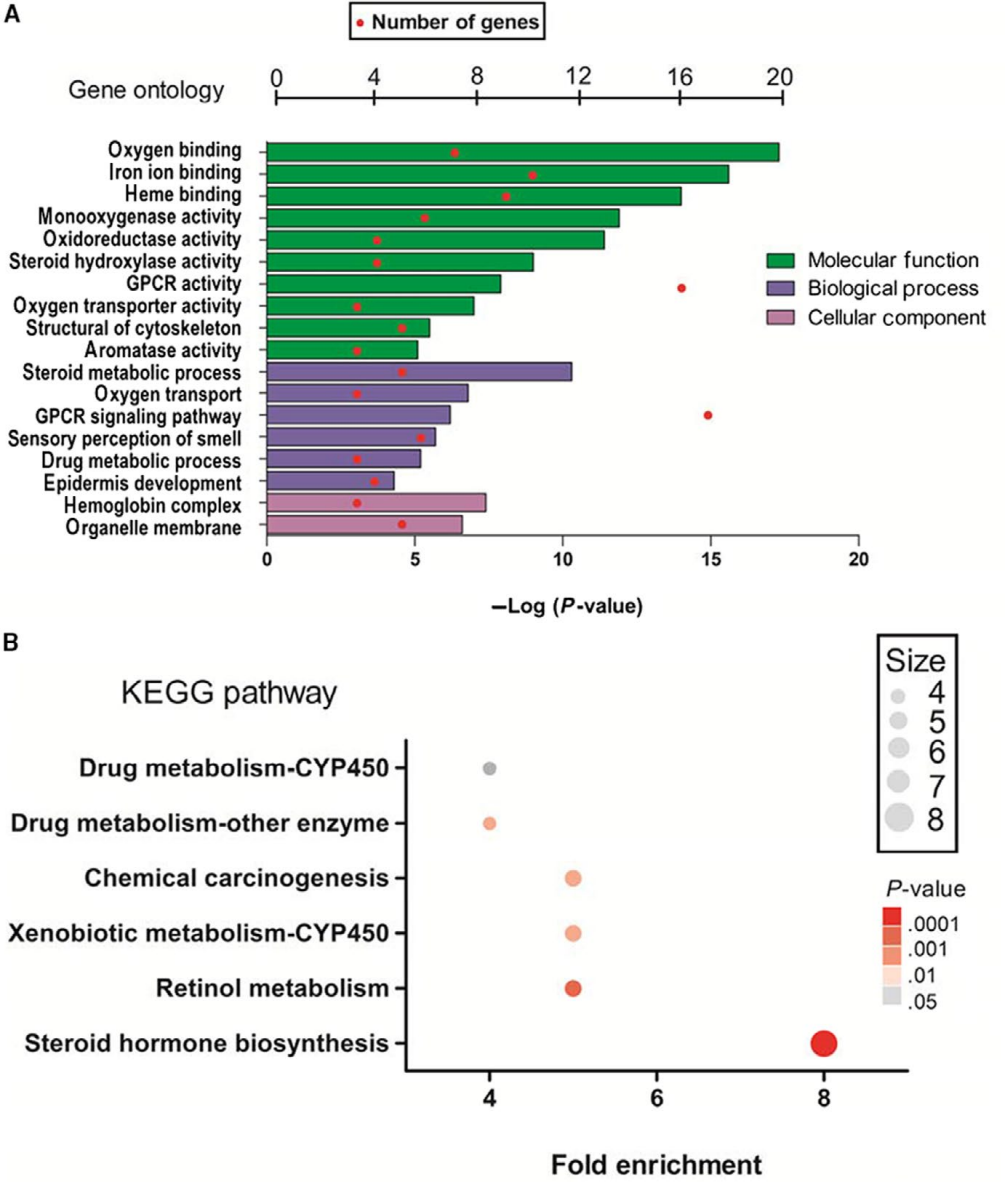
DAVID analysis of DEGs: A, GO functional annotation of top 18 enrichment terms. B, KEGG pathway enrichment analysis of top 6 enrichment terms. The count of genes enriched in terms is indicated by the node size; the *P* value is shown by the color, the redder the color, the more significant it is. DAVID, Database for Annotation, Visualization, and Integrated Discovery; DEGs, differentially expressed genes; GO, gene ontology; KEGG, Kyoto Encyclopedia of Genes and Genomes

Based on the DAVID software, a total of 18 GO functions were enriched. Concerning the Molecular Function terms, the 202 DEGs were mostly enriched in oxygen binding, iron ion binding, heme binding, monooxygenase activity, oxidoreductase activity, steroid hydroxylase activity, G protein‐coupled receptor (GPCR) activity, oxygen transporter activity, structure of the cytoskeleton, and aromatase activity. Concerning the Biological Process terms, the DEGs were significantly enriched in the steroid metabolic process, oxygen transport, the GPCR signaling pathway, the sensory perception of smell, the drug metabolic process, and epidermis development. Concerning the Cellular Component terms, the DEGs were significantly enriched in hemoglobin complex and organelle membrane (Figure 4A; Table 3).

**Table 3:** The 18 most significant enriched gene sets for recurrence features of stage II CRC from BP, MF, CC

ID	Description	Count	*P*‐Value
Molecular Function			
GO:0019825	Oxygen binding	7	6.14E‐06
GO:0005506	Iron ion binding	10	1.96E‐05
GO:0020037	Heme binding	9	6.04E‐05
GO:0004497	Monooxygenase activity	6	2.53E‐04
GO:0016712	Oxidoreductase activity	4	3.83E‐04
GO:0008395	Steroid hydroxylase activity	4	.00202095
GO:0004930	G‐protein coupled receptor activity	16	.00415544
GO:0005344	Oxygen transporter activity	3	.00799984
GO:0005200	Structural constituent of cytoskeleton	5	.02284408
GO:0070330	Aromatase activity	3	.02840488
Biological Process			
GO:0008202	Steroid metabolic process	5	7.68E‐04
GO:0015671	Oxygen transport	3	.00894403
GO:0007186	G protein‐coupled receptor signaling pathway	17	.01335430
GO:0007608	Sensory perception of smell	6	.01900123
GO:0017144	Drug metabolic process	3	.02772735
GO:0008544	Epidermis development	4	.04905401
Cellular Component			
GO:0005833	Hemoglobin complex	3	.00574369
GO:0031090	Organelle membrane	5	.01005061

Abbreviations: CRC, colorectal cancer; GO, gene ontology.

In KEGG pathway enrichment analysis, the 202 DEGs were significantly enriched in six signaling pathways: steroid hormone biosynthesis, retinol metabolism, xenobiotic metabolism‐CYP450, chemical carcinogenesis, drug metabolism‐other enzyme, and drug metabolism‐CYP450 (Figure 4B; Table 4).

**Table 4:** The 6 most significant enriched pathways for recurrence features of stage II CRC from KEGG

KEGG pathway id	Description	Count	*P*‐Value
hsa00982	Drug metabolism—cytochrome P450	4	.04376667
hsa00983	Drug metabolism—other enzymes	4	.01587008
hsa05204	Chemical carcinogenesis	5	.01355137
hsa00980	Metabolism of xenobiotics by cytochrome P450	5	.0103847
hsa00830	Retinol metabolism	5	.00660703
hsa00140	Steroid hormone biosynthesis	8	4.00E‐06

Abbreviations: CRC, colorectal cancer; KEGG, Kyoto Encyclopedia of Genes and Genomes.

### 3.6 PPI network construction and hub gene identification

**Figure 5:**
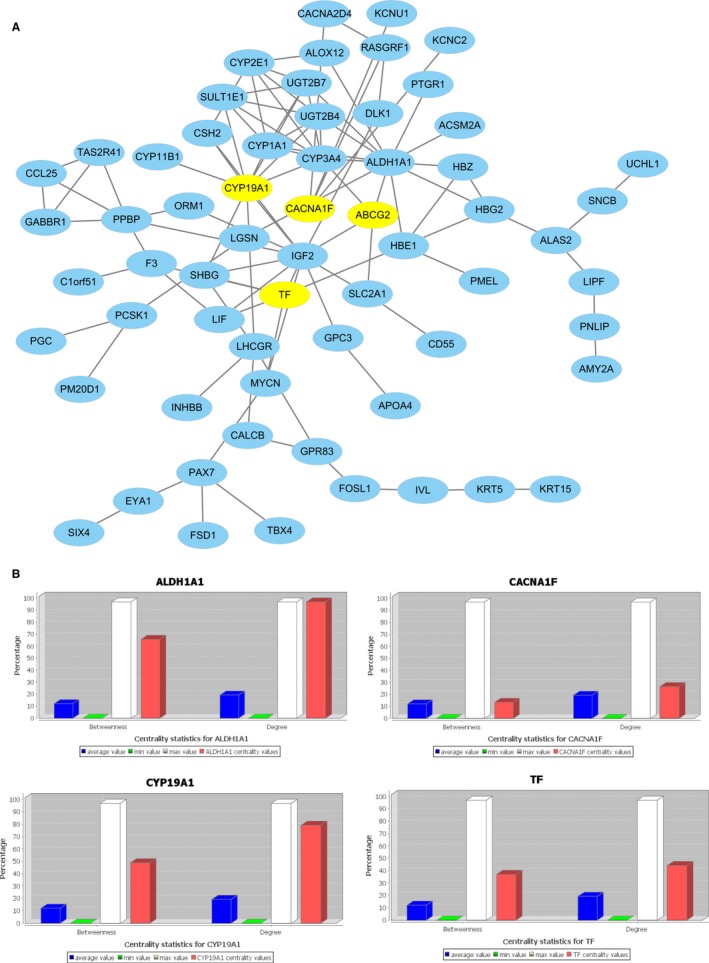
PPI Network of DEGs in recurrence compared nonrecurrence. The nodes indicate the DEGs and the edges indicate the interactions between two genes. The yellow nodes indicating important were selected as hub genes. A, The hub genes identified and visualized by degree and betweenness. B, Centrality statistics for hub genes: *ABCG2*, *CACNA1F*, *CYP19A1*, *TF*. DEGs, differentially expressed genes; PPI, protein‐protein interaction

Based on the STRING database, a PPI network was constructed (Figure 5A). The network contained 63 nodes and 100 edges that were subjected to hub gene analysis with CentiScaPe 2.2. Four hub genes evaluated by the degree (≥3.14) and betweenness (≥223) were identified: *ABCG2*, *CACNA1F*, *CYP19A1*, and *TF* (Figure 5B; Table 5).

**Table 5:** The four hub genes in the protein‐protein interaction network

Gene	Description	Degree	Betweenness
*ABCG2*	ATP ‐binding cassette subfamily G member 2	4.0	477.4
*CACNA1F*	Calcium voltage‐gated channel subunit alpha1 F	4.0	242.0
*CYP19A1*	Cytochrome P450 family 19 subfamily A member 1	10.0	899.3
*TF*	Transferrin	6.0	680.8

### 3.7 The clinical value of the four hub genes in all stages of CRC

**Figure 6:**
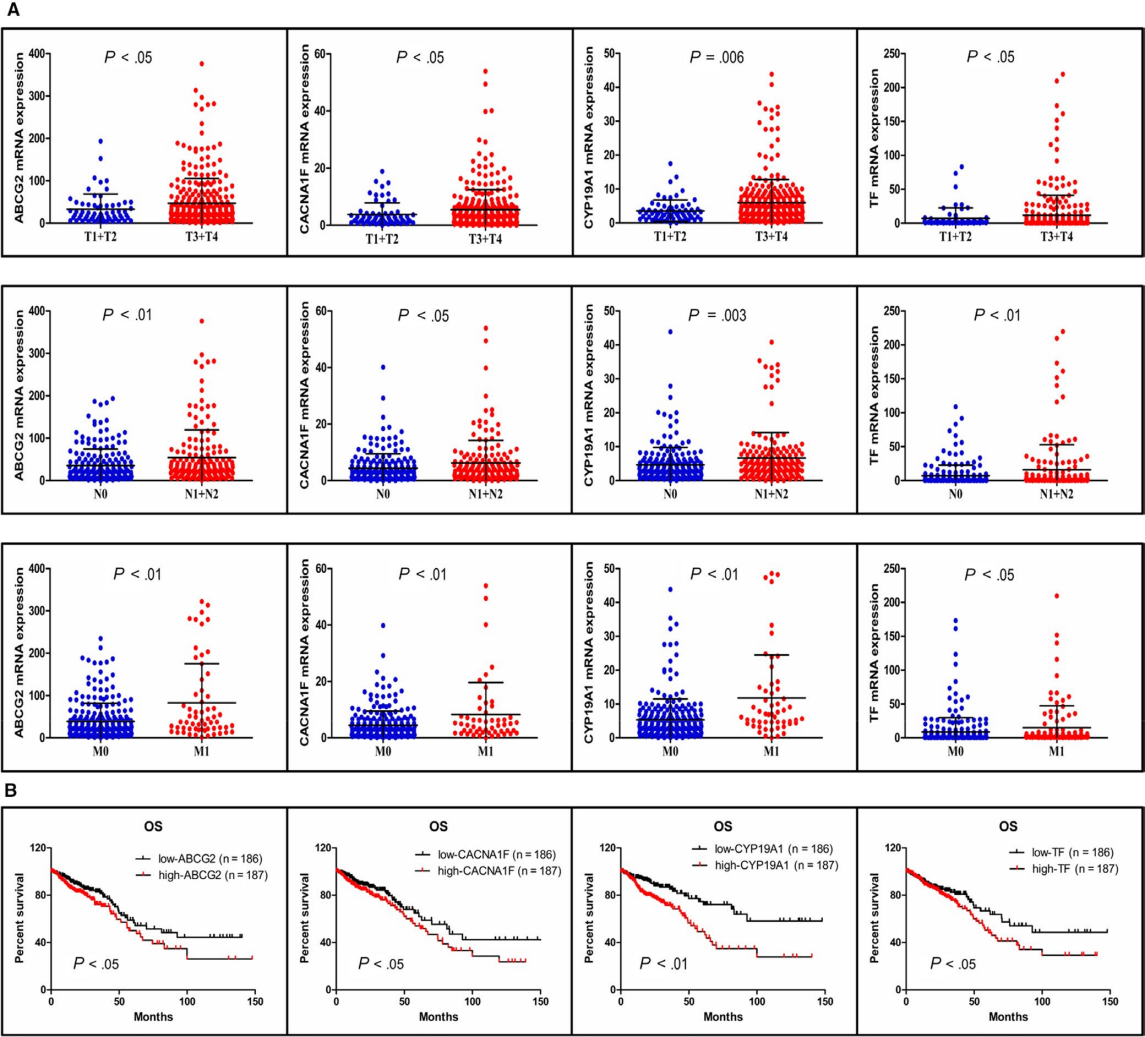
Clinical values of the four hub genes in all stage CRC. A, Validation of the gene expression levels of *ABCG2*, *CACNA1F*, *CYP19A1*, *TF* in different TNM stages patients. B, OS analysis of the four hub genes. CRC, colorectal cancer; OS, overall survival

Using the TCGA transcriptional profiles in all stages of CRC, the expression levels of the four hub genes with different TNM stages were significantly different, and the high expression of *ABCG2*, *CACNA1F*, *CYP19A1*, and *TF* was associated with a poor TNM stage (Figure 6A). For the T stage (T_1+2_ vs T_3+4_), the *P* values of *ABCG2*, *CACNA1F*, *CYP19A1*, and *TF* were <.05, <.05, .006, and <.05, respectively. For the N stage (N_0_ vs N_1+2_), the *P* values of *ABCG2*, *CACNA1F*, *CYP19A1*, and *TF* were <.01, <.05, .003, and <.01, respectively. For the M stage (M_0_ vs M_1_), the *P* values of *ABCG2*, *CACNA1F*, *CYP19A1*, and *TF* were <.01, <.01, <.01, and <.05, respectively. The high expression of *ABCG2*, *CACNA1F*, *CYP19A1*, and *TF* was also associated with poor OS (Figure 6B), with *P* values of <.05, <.05, <.01, and <.05, respectively.

## 4 DISCUSSION

In our quest to develop a robust recurrence model of stage II CRC, we successfully developed the GRM, which achieved excellent predictive values of recurrence in stage II CRC. The underlying mechanisms of recurrence were also explored.

Current high‐risk criteria for defining the recurrence of stage II CRC, which were addressed in the introduction, depend largely on clinicopathologic factors, leading to limitations in their prognostic abilities for this highly heterogeneous tumor, and the improvement in stage II CRC patient survival following adjuvant chemotherapy is less than 5% at 5 years.^15^ This means that the administration of adjuvant chemotherapy to all patients in stage II is approximately 75% unnecessary and harmful. This narrow therapeutic index underscores the importance of identifying more appropriate biomarkers to detect the genuine high‐risk factors of recurrence for stage II CRC.

Single biomarkers, such as *CEA*, *CA199*, *miR‐21*, *miR‐181c*, *MTA3*, *S100A2*, and *ezrin*,^5^, ^16^, ^17^, ^18^, ^20^, ^21^ for the prognosis of recurrence in stage II CRC patients have been reported before. However, given that CRC tissues show complicated molecular and cellular heterogeneity, a single biomarker failed to reflect the genomic heterogeneity of the tumor; therefore, their prediction efficiency was unpowerful. Although multigene expression signatures have been reported, the number of genes that need to be tested is 13, 31, or 120, which is uneconomical, and the signatures demonstrated poor feasibility and poor specificity.^22^, ^23^, ^24^ One previously reported 8‐miRNA recurrence classifier is superior to currently used clinicopathological features, as well as NCCN criteria. Another prognostic mutation panel comprising five prognostic genes (*APAF1*, *DIAPH2*, *NTNG1*, *USP7*, and *VAV2*)^25^ also showed superior prognostic accuracy to that of the American Joint Commission on Cancer classification (concordance index: 0.70 vs 0.54, respectively). However, the authors showed only the HR; therefore, its specific diagnostic performance is unclear. Miyake et al constructed a discriminator gene set that included 30 genes based on the expression data of 92 stage II CRC patients; however, despite its reported diagnostic effectiveness, its prediction accuracy was only 77.4%.^26^

Under these circumstances, we explored the prediction efficiency of expression datasets from the TCGA database of stage II CRC patients who did not undergo postoperative adjuvant therapy and were followed up for at least 2 years. Using random forest variable hunting, we identified the top 5 DEGs. As the results revealed, different combinations of the top 5 DEGs showed different AUC values. The combination of the four‐gene signature (*ZNF561*, *WFS1*, *SLC2A1*, and *PTGR1*), with an AUC of 0.882, exhibited the best performance for predicting recurrence and showed remarkable sensitivity and specificity when the cutoff value was 0.593. Therefore, these four genes were selected to create the GRM and achieved excellent predictive values of recurrence, with an AUC of 0.882 in stage II CRC. To further test and verify the diagnostic effectiveness of the GRM, we extracted another gene expression profiling dataset from the GEO (GSE12032) for further validation. To our excitement, all the genes in the GRM were included in the top 10 DEGs ranked by the MDG with the random forest classifier (Table 2) from the GEO database and exhibited better performance for predicting recurrence, with an AUC of 0.943, than the TCGA database. Furthermore, we and Miyake et al used the same gene expression profiling dataset. Our GRM contains only four genes but exhibited robust performance for predicting recurrence, with an AUC of 0.943, while the prediction accuracy of their discriminator gene set, which contains 30 genes, was only 77.4%. However, the other genes selected before by different teams for the prediction of recurrence in stage II CRC were obtained using different methods; therefore, their diagnostic performance cannot be directly compared with ours. However, the main methods we used to select the DEGs to establish the GRM were advanced, such as the random forest method, which takes advantage of two powerful machine‐learning techniques: bagging and random feature selection. As one of the most important advantages, accuracy mainly benefits from the complementation of the training set and the test set and is relatively robust to outliers and noise, which contribute to its superior performance over many other methods, and it is commonly used in genomic data analyses as an effective prediction tool.^27^, ^28^

The robust diagnostic effectiveness showed by the GRM in two independent databases highly emphasized its stability. Stability mainly depends on the high efficiency, robustness and reliability of the analytical methods we used, such as the random forest method and ROC curve analysis. Furthermore, the robust diagnostic effectiveness also encouraged us to further apply it in clinical practice, and the personalized prediction of recurrence by the GRM will help avoid undertreatment or overtreatment.

Further exploration of the clinical values of the four genes in the GRM validated their effectiveness in the diagnosis of recurrence in all stages of CRC. The low expression of *ZNF561* and *PTGR1* and the high expression of *WFS1* and *SLC2A1* were associated with poor DFS. All of the above findings strongly prove the high recurrence diagnostic efficiency of our GRM.

To explore the mechanism of recurrence in stage II CRC, we used an integrated bioinformatics analysis. In GO analysis, 18 functions were enriched and support evidence for the important roles of oxygen, epidermis development, hemoglobin, and GPCR.

Hypoxia (low oxygen concentration) and ischemia (low hemoglobin concentration), which are caused by insufficient vascularization, are hallmarks of solid tumors (including CRC).^29^, ^30^ Hypoxia acts as an off switch for the expression of several genes, such as vascular endothelial growth factor (*VEGF*) and epidermal growth factor receptor (*EGFR*),^31^, ^32^ and could facilitate tumor development by epithelial‐to‐mesenchymal transition^33^, ^34^; moreover, intratumoral hypoxia is associated with therapy resistance, metastasis, and a poor clinical outcome in CRC.^34^ Under conditions of hypoxia, the transcription factor hypoxia‐inducible factor (*HIF*)^35^ is activated immediately and strongly adapts to the environment by regulating transcriptional programs in erythropoiesis, angiogenesis and metabolism.^36^ Then, it secretes a large number of angiogenesis‐related molecules, such as *VEGF*, to actualize angiogenesis, cell proliferation and protection against ischemic injury.^37^

Heme has important functions in transportation, catalysis, and electron and signaling transfer and serves as a prosthetic group in heme binding. Heme binding is widely observed in tumor lesions and cancer cells, and heme‐binding ability is necessary for DNA damage resistance^38^ and represents an important biomarker of the proliferative status of cancers.^39^ Hemoglobin, which is the most abundant heme‐binding protein associated with oxygen transportation,^40^ indicates hypoxia, and the heme‐binding ability and hemoglobin yield exhibit correlative dependence and interplay.

G protein‐coupled receptors comprise the largest superfamily of receptors involved in transmembrane‐initiated transduction pathways and can crosstalk (or transactivate) with *EGFR*, insulin/insulin‐like growth factor 1 receptors and other cell surface receptors^41^ to mediate the proliferation,^42^ angiogenesis,^43^ and metastasis^44^ of CRC.

Among the mechanisms involved in the above biological processes, oxygen binding,^45^ iron ion binding,^45^ heme binding, oxygen transporter activity, oxygen transport, the hemoglobin complex, epidermis development, and the GPCR signaling pathway,^46^ which were highlighted in the results of the GO functional annotation, are crucial in tumor development; therefore, targeting *HIF*, *VEGF*, iron ion binding, and GPCR may be good options for preventing the recurrence of stage II CRC.

In KEGG analysis, the main enrichment pathways were xenobiotic metabolism (including drug and retinol)‐CYP450/other enzymes, chemical carcinogenesis and steroid hormone biosynthesis.

Xenobiotic metabolism involves the metabolism of potentially harmful compounds that can enter the body together with food, environmental components, drugs or food additives. Xenobiotic metabolism enzymes include cytochrome P450 (CYP), the glutathione S‐transferase (GST) family, the uridine 50‐diphospho–glucuronosyltransferase (UDP‐glucuronosyltransferase‐UGT) superfamily, alcohol‐metabolizing enzymes, sulfotransferases, etc. Under normal circumstances, xenobiotic metabolism enzymes play the role of detoxification. However, they can also convert certain chemicals into highly toxic metabolites to trigger chemical carcinogenesis, which is known as ‘‘bioactivation’’.^47^ Different alleles of enzymes involved in xenobiotic metabolism contribute to CRC susceptibility^48^; for example, CYP450 can increase the metabolic activity of procarcinogens, which include polycyclic aromatic hydrocarbons and heterocyclic amines, resulting in the production of potential carcinogens and eventually the development of CRC.^49^ Another metabolic enzyme is GST, which plays a major role in detoxification and steroid hormone biosynthesis.^50^

Steroid hormone and its receptor have protective functions against the progression of CRC^51^; therefore, the expression of steroid hormone in colon cancer tissues is lower than that in normal tissues,^52^ and the downregulation of steroid hormone expression in colorectal tissues is a cancer signal.

Retinol inhibits CRC cell proliferation,^53^ even inhibiting invasion through a retinoic acid receptor‐independent mechanism.^54^ In vivo, an impairment in hepatic and intestinal cytosolic retinol oxidation and retinoic acid formation by alcohol abuse can increase the risk of developing CRC, and retinol metabolism plays a very important role in this process.^55^

Based on the above mechanisms of the enriched pathways, we hypothesized that the recurrence of stage II CRC is likely due to “the breach of duty” of the xenobiotic metabolism enzymes, leading to the decompensation of retinal metabolism and the inhibition of steroid biosynthesis, and ultimately the development of recurrence. The metabolism of other potentially harmful compounds may also be involved in regulating xenobiotic metabolism enzymes while avoiding the invasion of xenobiotics and supplementing retinal and steroid hormones, which may be good options for preventing the recurrence of stage II CRC.

To further highlight the hub genes that play the most important roles in recurrence and to explore their interactions, a PPI network was constructed, and four hub genes were selected. *ABCG2*, which is a membrane‐associated protein, can relieve oxidative stress and the inflammatory response by inhibiting the NF‐κB signaling pathway, and the high expression of *ABCG2* in CRC tissues may represent feedback of the overoxidative reaction, which is associated with a poor prognosis.^56^, ^57^ Most research has reported that inhibiting *ABCG2*, as a marker of chemoresistance in CRC, could enhance the efficacy of Hypericin‐mediated photodynamic therapy. *CACNA1F* is a voltage‐gated calcium channel that is mainly expressed in the human retina, but it has also been reported to be widely distributed outside the retina, including in the immune system.^58^ It is well documented that *CACNA1F* plays roles in cell proliferation, migration, and apoptosis,^59^ but it is rarely reported in CRC; therefore, further research on *CACNA1F* is needed. *CYP19A1* is a member of the CYP450 superfamily that, as a monooxygenase, catalyzes drug metabolism and the synthesis of cholesterol, steroids and other lipids. Many researchers suggest that polymorphisms in *CYP19A1* are related to CRC risk and may be influenced by estrogen through an inflammation‐related mechanism.^60^, ^61^ The main function of the *TF* protein is to transport iron from the intestine, reticuloendothelial system, and liver parenchymal cells to all proliferating cells in the body. *TF* is an iron‐transporting protein that can transfer the iron absorbed by the intestinal mucosa to the bone marrow for hemoglobin formation in normoblasts.^62^ A large number of studies indicate that *TF* is also a growth factor of all proliferated and cultured cells.^63^ Moreover, *TF* is synthesized for its own specific proliferation and differentiation in tumor tissue. Based on the biological properties of *TF*, *TF* in the feces is used as a blood marker for CRC screening, with a sensitivity and specificity of 92% and 72.0%, respectively.^64^, ^65^

However, our current study has the following limitations: (a) Because we were unable to obtain complete clinical data, we were unable to implement a comparison of predictive effectiveness between our GRM and high‐risk factors defined by the NCCN guidelines. (b) Our study is based on a mRNA evaluation from the TCGA and GEO databases; therefore, it is not as persuasive as the level of protein expression, and we did not use clinical samples to further verify its authenticity. (c) It is a retrospective study; therefore, the evidence level is imperfect. With regard to potential limitations, our GRM and hub genes relying on the advantages of excellent predictive values and reasonable statements should be validated in future, prospective, multicenter clinical trials. In addition, biomarkers that show promising predictive value for the survival benefit of adjuvant chemotherapy in stage II CRC patients should be discovered at the same time.

In summary, our findings showed that the GRM can effectively classify stage II CRC patients into groups with high and low risks of recurrence, thereby making up for the prognostic value of the traditional clinicopathological risk factors defined by the NCCN guidelines. Moreover, various pathways and hub genes involved in the recurrence progression of stage II CRC were revealed, which may be useful therapeutic targets. Thus, the GRM and hub genes could offer clinical value in directing individualized and precision therapeutic regimens for stage II CRC patients.

## 5 CONCLUSION

In conclusion, the GRM we established using stage II CRC data from the TCGA by random forest variable hunting showed robust diagnostic effectiveness and was further validated with GEO data, supporting its robust ability in the personalized prediction of recurrence. In addition, GO, KEGG, PPI network analyses, and hub gene selection revealed the underlying mechanism of recurrence to a certain extent. Thus, the GRM and hub genes could offer clinical value in directing individualized and precise therapeutic regimens for stage II CRC patients.

## CONFLICT OF INTEREST

The authors report no conflict of interest in this work.

## Data Availability

The datasets used and/or analyzed in current study can be obtained from the corresponding author on reasonable request.
